# The Mediterranean-dietary approaches to stop hypertension diet intervention for neurodegenerative delay (MIND) diet: a bibliometric analysis

**DOI:** 10.3389/fnut.2024.1348808

**Published:** 2024-06-14

**Authors:** Linxiong Dai, Xiaoxiao Lin, Shuai Wang, Yue Gao, Fei He

**Affiliations:** ^1^Taizhou Municipal Hospital, Taizhou, China; ^2^Hangzhou First Hospital, Hangzhou, China

**Keywords:** MIND diet, cognitive function, dementia, bibliometric analysis, Alzheimer’s disease

## Abstract

The MIND diet is a healthy dietary pattern that has some benefits for many health outcomes. Our study aims to conduct a bibliometric analysis of the MIND diet, identifying leading edges and hotspots to provide a reference for future research. The research on the MIND diet was gathered from the Web of Science Core Collection (WOSCC) database. For bibliometric analysis, VOSviewer 1.6.16 and the WOSCC Online Analysis Platform were utilized. In total, this comprehensive investigation encompassed 171 documents in the field of the MIND diet. The publications are globally distributed, with contributions from 953 authors across 362 institutions in 37 countries/regions, and published in 94 journals. The United States leads with 72 publications, and Iran and the People’s Republic of China also show notable engagement with 28 and 19 publications, respectively. Rush University stands out with 21 publications, followed by Harvard University and Tehran University of Medical Sciences, demonstrating their substantial contributions to this field. Martha Clare Morris is a key figure with 10 publications, alongside Klodian Dhana and Puja Agarwal, each contributing 9 publications, highlighting their influence in the MIND diet research. The journal “Nutrients” is a major publication venue with 20 related articles, followed by “Frontiers in Nutrition” and “Journal of Nutrition Health Aging,” reflecting their crucial roles in advancing knowledge about the MIND diet. The first high-cited publication was published in *Alzheimers & Dementia* and conducted by Martha Clare Morris, which focuses on the MIND diet’s relationship with Alzheimer’s disease prevention and cognitive decline and emphasizes the diet’s neuroprotective potential, highlighting how even moderate adherence can substantially reduce Alzheimer’s risk and slow cognitive decline. In conclusion, this is the first comprehensive bibliometric study that quantitatively and qualitatively analyzed the publications in the field of the MIND diet. The MIND diet may be a promising dietary pattern for dementia. However, the current evidence is restricted and highlights the urgency and necessity of further research to investigate the efficacy of this diet for cognitive function. In addition, the MIND diet may have some benefits for other health outcomes, including CVDs, cancer, and diabetes. The number of studies in the field of the MIND diet is limited. More studies are needed, and will give us more knowledge about the MIND diet to improve human health, especially for dementia.

## 1 Background

The MIND diet is a nutritional plan combining the Mediterranean and Dietary Approaches to Stop Hypertension (DASH) diets (1–6). It’s specifically tailored to reduce the risk of neurodegenerative diseases like Alzheimer’s and other dementias. This diet emphasizes brain-healthy foods such as leafy greens, berries, nuts, whole grains, and fish. It inherits the heart-healthy attributes of both the Mediterranean and DASH diets. The MIND diet restricts unhealthy food intake, advising limited consumption of red meat, butter and margarine, cheese, pastries, sweets, and fried or fast food. It also endorses moderate wine drinking, particularly red wine, for its supposed brain health benefits. Created by nutritional epidemiologists at Rush University in Chicago, the MIND diet is based on research into the neuroprotective qualities of specific foods and diets. Studies (2, 5) indicate that following the MIND diet, even to a moderate extent, correlates with a lower risk of Alzheimer’s and slower cognitive decline. Some diets such as MIND diet, Mediterranean Diet, Anti-inflammatory diet, and Ketogenic Diet plays a significant role in the management and potentially in the prevention of neurodegenerative diseases such as Alzheimer’s disease, Parkinson’s disease, and multiple sclerosis. Research in this area focuses on how certain foods and dietary patterns can influence brain health over time. Distinguishing itself from the Mediterranean and DASH diets (5), which focus more on cardiovascular health, the MIND diet zeroes in on brain health. It targets foods rich in antioxidants and nutrients that fight oxidative stress and inflammation, major factors in neurodegenerative diseases. The MIND diet stands at the intersection of nutrition and neurology, proposing a diet-based strategy to delay or prevent neurodegenerative diseases by emphasizing brain-healthy foods. This approach highlights the significant impact of diet on both physical and cognitive health.

The MIND diet, gaining attention for its role in neurodegenerative disease prevention, lacks a comprehensive bibliometric analysis. This is crucial for summarizing publication trends and identifying research hotspots. Bibliometric analysis (7–9), a systematic, quantitative method, evaluates scholarly literature on topics like the MIND diet. It involves collecting and assessing data on publications, citations, authors, journals, and institutions, providing insights into research trends, knowledge networks, and the diet’s impact. This approach helps identify key patterns, influential contributors, and evolving research themes, and spot emerging areas and gaps in literature. Useful for academics and policymakers, it offers a comprehensive view of the scientific discourse and knowledge dissemination in diet and cognitive health. Our study aims to conduct a comprehensive bibliometric analysis of the MIND diet, identifying leading edges and hotspots to provide a reference for future research.

## 2 Methods

### 2.1 Data sources and search strategies

For this bibliometric study, the publications on the MIND diet were gathered from the Web of Science Core Collection (WOSCC) database. The search terms used for this analysis included “MIND diet*,” “MIND diets,” “MIND dietary pattern,” “Mediterranean-DASH,” and “Mediterranean-Dietary Approaches to Stop Hypertension Diet Intervention for Neurodegenerative Delay,” focusing on research published up to October 15, 2023. The study included all publications related to the MIND diet.

### 2.2 Bibliometric analysis and visualization

In the bibliometric analysis and visualization stage, various metrics from the selected literature were examined using the WoSCC Online Analysis Platform. This analysis covered the yearly publication count, identification of the top 10 most active countries/regions, institutions, authors, and journals, along with the top 20 most-cited articles related to “the MIND diet” topic. For a detailed examination, the data was downloaded in TXT format with full records and cited references. The VOSviewer software (version 1.6.16) was then employed to create visualizations of different datasets. These visualizations included co-authorship networks across institutions, countries/regions, and individual authors, journal and reference citation patterns, co-citation networks, and keyword co-occurrence networks. The final step involved exporting relevant visualizations to support and illustrate the findings of this bibliometric analysis. Bibliometric indicators are quantitative tools used to evaluate the impact and relevance of scholarly publications. Key indicators include the citation count, which tracks how many times a publication has been cited by other works, and the h-index, which measures a researcher’s productivity and citation impact by ensuring that an author has h papers each cited at least h times. Collectively, these indicators help assess the quality and influence of scientific research across different disciplines and media.

## 3 Results

### 3.1 Trends in global publications

**Figure 1:**
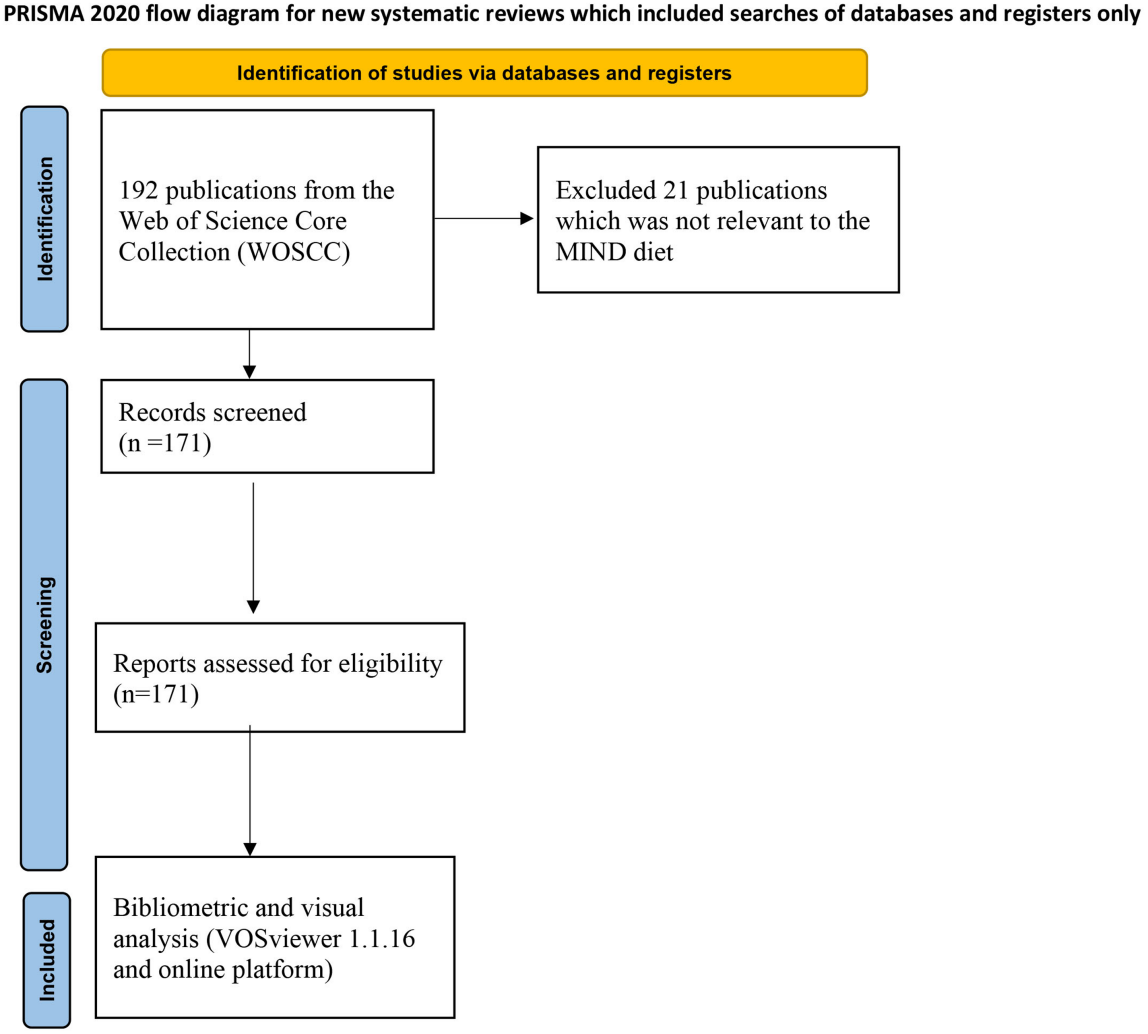
Flowchart of the inclusion and exclusion criteria on publications of MIND diet.

**Figure 2:**
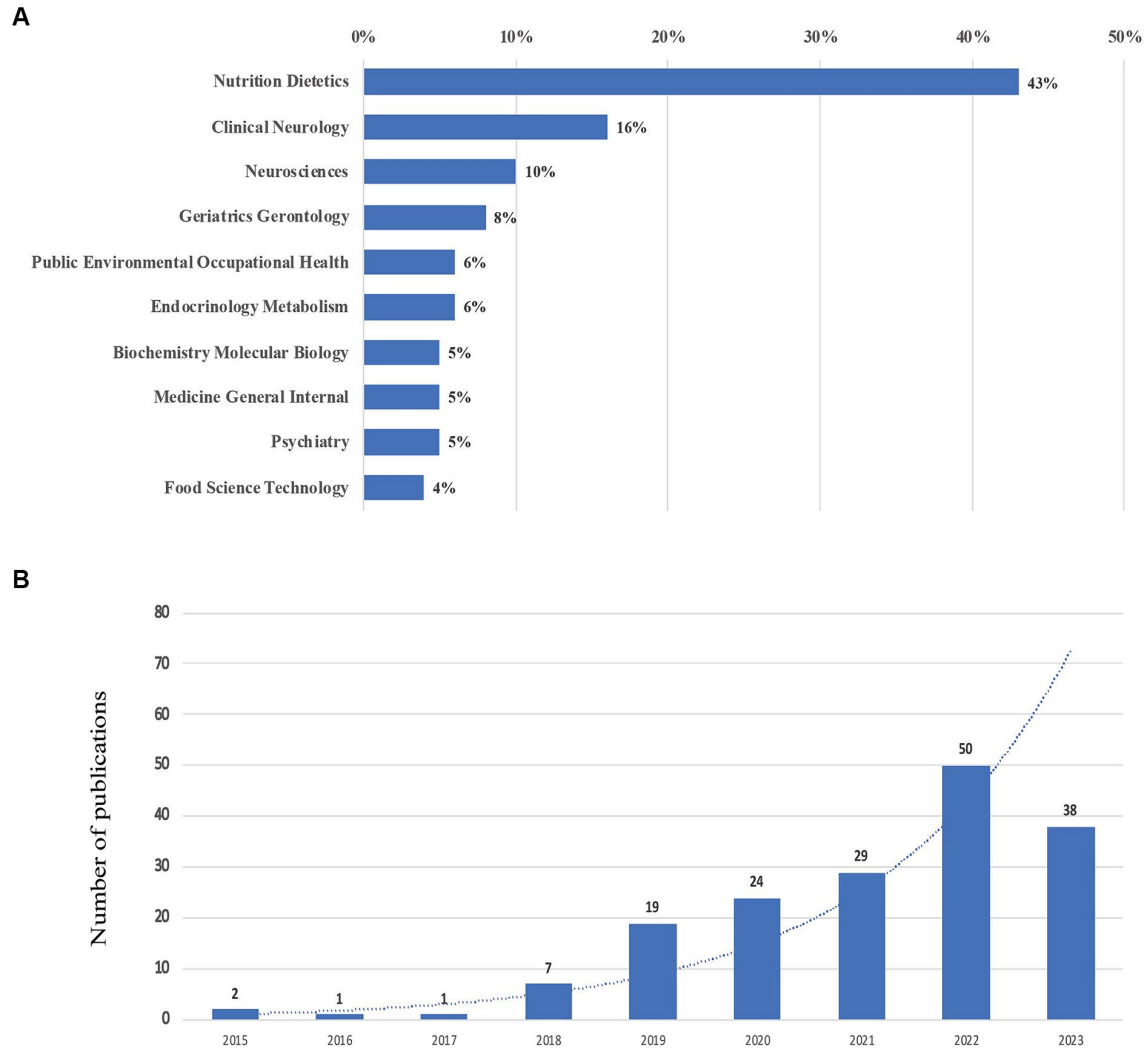
Yearly quantity and literature type of publications on the MIND diet from inception to Oct 15, 2023. **(A)** Literature type distribution. **(B)** Annual publication quantitative distribution.

Based on the provided visual data, a total of 171 documents concerning the topic of MIND diet were identified in the bibliometric study, which is shown in Figure 1. For the types of 171 publications, there were 49 reviews, 51 cohort studies, 65 cross-sectional studies, and 6 randomized control trials (RCTs). Most publications (119/171, 69.6%) are focused on the dementia, some publications (21/171, 12.3%) on Parkinson’s disease, and the remaining publications (31/171, 18.1%) on other neurodegenerative disorders including unspecific focus, schizophrenia and depression. The bar chart shows the proportion of publications across various scientific fields. ‘Nutrition Dietetics’ is the most represented category, accounting for 43% of the publications, which aligns with the MIND diet’s focus on nutritional interventions for cognitive health. Notably, ‘Clinical Neurology’ and ‘Neurosciences’ make up 16 and 10% respectively, suggesting significant research interest in the neurological and cognitive outcomes associated with dietary patterns. The inclusion of fields like ‘Geriatrics Gerontology’ and ‘Endocrinology Metabolism’ at 8 and 6% respectively, may reflect the MIND diet’s relevance to aging populations and metabolic health. This graph illustrates the growth in the number of publications from 2015 to 2023. There is a clear upward trajectory in the volume of research, with a particularly steep increase from 2021 to 2022. This pattern suggests expanding interest and investigation into the field, potentially highlighting the growing recognition of diet’s role in mental and cognitive health, areas central to the MIND diet’s principles. In summary, the bibliometric data suggests a burgeoning interest in the intersection of nutrition and cognitive health, with the MIND diet likely being a key area of focus given the fields represented. The rapid increase in publications in recent years highlights the diet’s rising prominence in research and its potential implications for public health, particularly regarding aging and cognitive function. The yearly quantity and literature type of publications are shown in Figure 2.

### 3.2 Countries/regions, academic institutions, authors, and journals analysis

**Figure 3:**
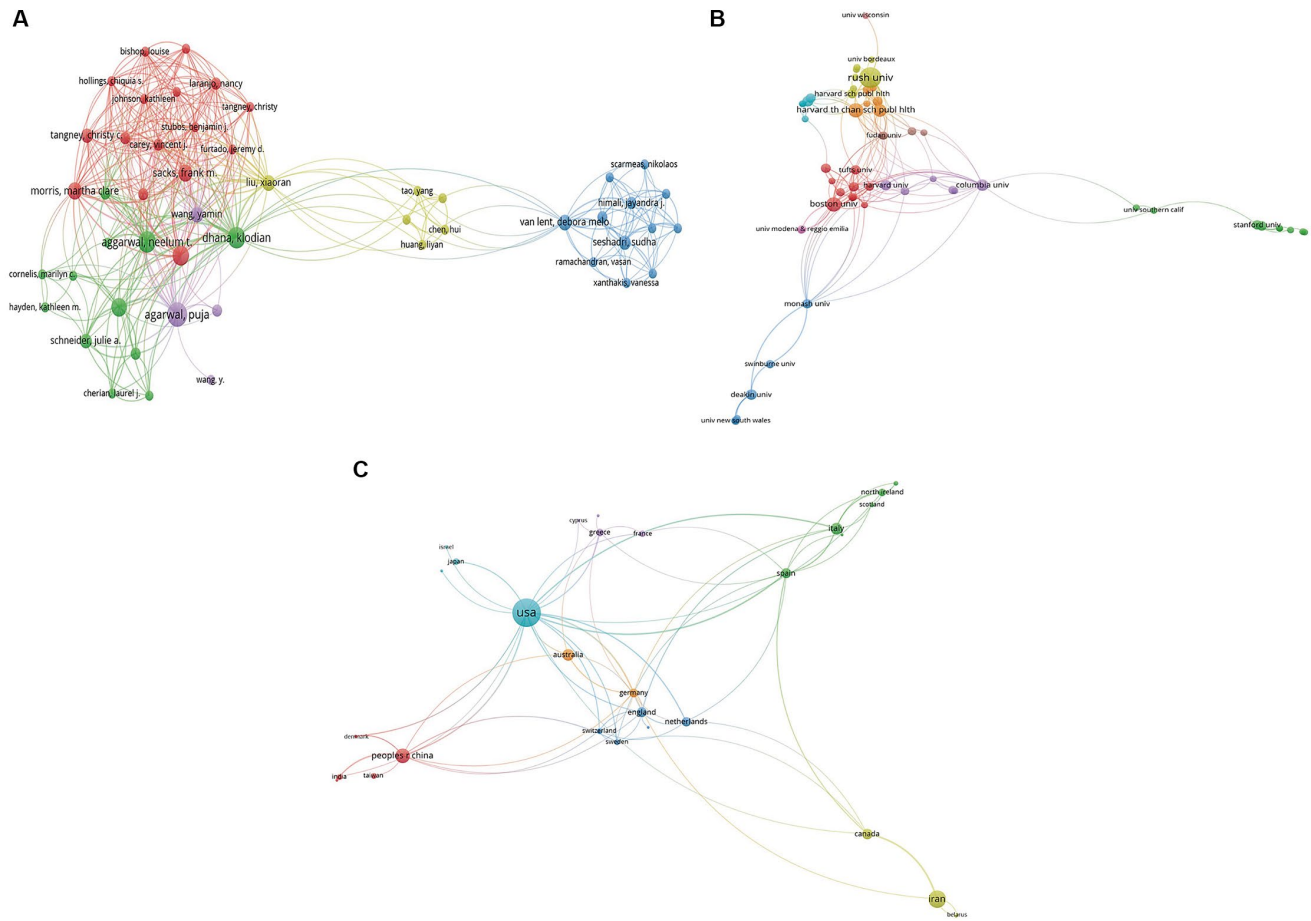
Visualization knowledge maps of authors, institutions, and countries/regions on the MIND diet. **(A)** The co-authorship map of authors. **(B)** The co-authorship map of institutions. **(C)** The co-authorship map of countries/regions.

**Figure 4:**
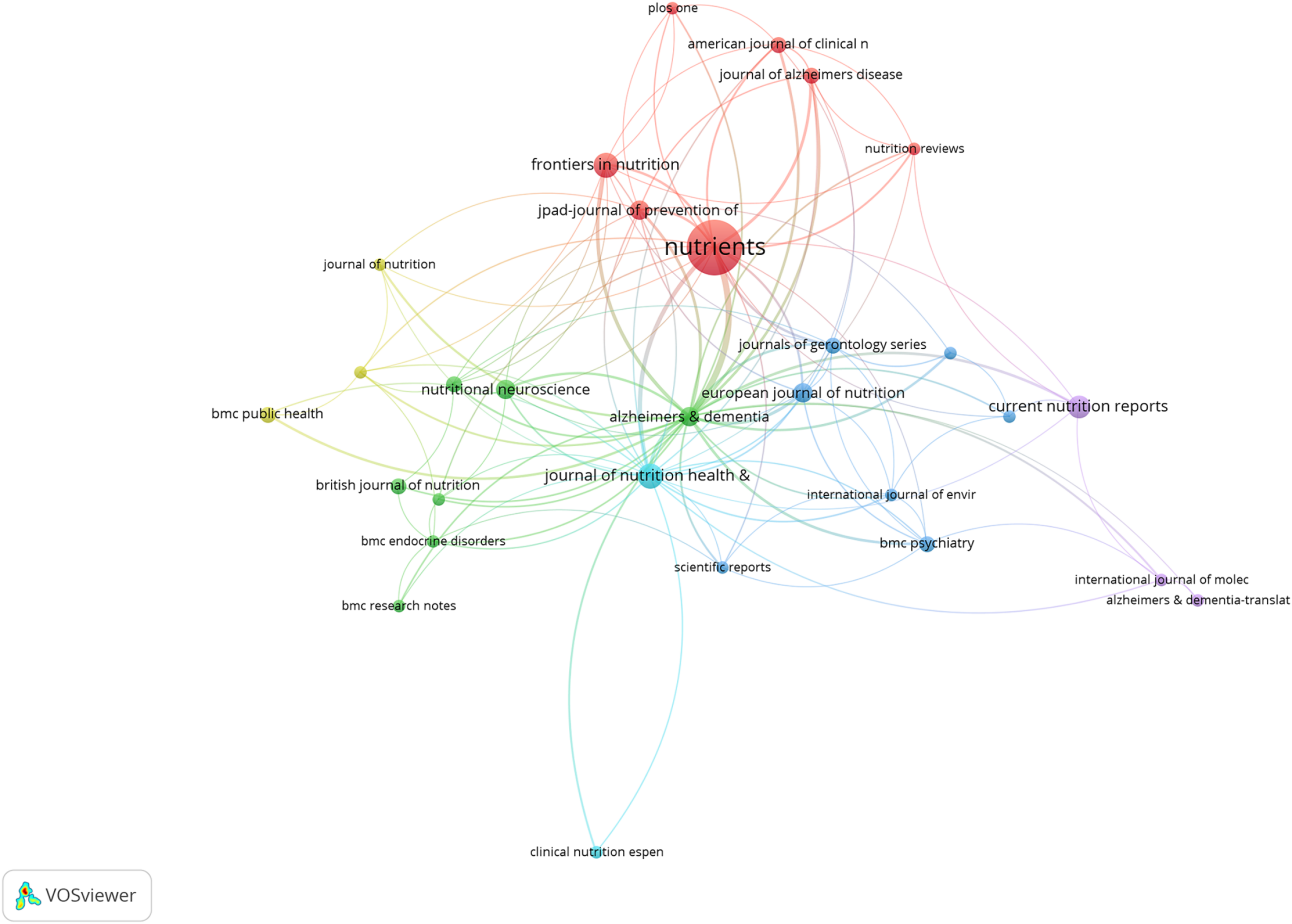
The visualization knowledge map of journals on the MIND diet.

In total, 953 authors from 362 institutions and 37 countries/regions with the publications in 94 journals contributed to the field of MIND diet. The bibliometric analysis indicates that the USA leads in research publications on the MIND diet with 72 publications. Iran, with 28 publications, and the People’s Republic of China, with 19, also display a notable interest, pointing to a global research engagement with the MIND diet. Rush University is the most prolific institution with 21 publications on the MIND diet, marking it as a central research hub in this area. Harvard University follows with 19 publications, and Tehran University of Medical Sciences with 15, showing their significant academic input. Martha Clare Morris emerges as a key researcher with 10 publications on the MIND diet, indicating her central role in the field. Klodian Dhana, with 9 publications, and Puja Agarwal, with 9 publications as well, are also significant contributors to the research on the MIND diet. The journal “Nutrients” leads with 20 publications concerning the MIND diet, emphasizing its prominence as a crucial outlet for research dissemination. “Frontiers in Nutrition” and “Journal of Nutrition Health Aging” follow, with 6 and 6 publications respectively, highlighting their roles in advancing the scholarly conversation about the MIND diet. In summary, the bibliometric analysis underscores the substantial role of the USA in country-specific and institutional contributions to MIND diet research. Distinguished researchers such as Martha Clare Morris have made considerable contributions, and publications like “Nutrients” are essential in spreading research findings. This reflects the increasing scientific focus on the MIND diet within the international community. Tables 1, 2 summarize the leading authors, institutions, and countries/regions contributing to the field of the MIND diet. Figures 3, 4 display network visualization maps illustrating the citation relationships among countries/regions, institutions, authors, and journals in this area of study. The top three cooperative authors were Klodian Dhana, Puja Agarwal, and Neelum T Aggarwal. The top three cooperative institutions were Rush University, Boston University, and Harvard University. The top three cooperative countries were the USA, Spain, and Germany. The top three cooperative journals were Alzheimers Dementia, Nutrients, and Journal of Nutrition Health Aging.

**Table 1:** The top 10 productive authors, institutions and countries based on publications of MIND diet.

Items	Publications			
	Rank	Country	Number	Citations
Country	1	USA	72	1,676
	2	Iran	28	209
	3	Peoples R China	19	126
	4	Italy	13	582
	5	Australia	12	195
	6	Canada	10	107
	7	England	9	365
	8	Spain	9	223
	9	Netherlands	8	350
	10	Germany	7	75
Institution	1	Rush University	21	1,180
	2	Harvard University	19	1,178
	3	Tehran University of Medical Sciences	15	109
	4	Boston University	9	76
	5	University of California System	9	119
	6	Isfahan University Medical Science	8	71
	7	Ciber Centro De Investigacion Biomedica En Red	7	103
	8	Shahid Beheshti University Medical Sciences	7	39
	9	University of Texas Health Science Center At San Antonio	7	68
	10	Columbia University	6	47
Author	1	Martha Clare Morris	10	1,067
	2	Klodian Dhana	9	111
	3	Puja Agarwal	9	144
	4	Neelum T. Aggarwal	9	1,004
	5	David A. Bennett	7	1,003
	6	Ahmad Esmaillzadeh	6	70
	7	Frank M. Sacks	6	952
	8	Debora Melo van Lent	6	55
	9	Lisa L. Barnes	6	513
	10	Thomas M. Holland	5	105

**Table 2:** The top 10 most productive journals on MIND diet.

Ranking	Journal name	Country	Counts
1	Nutrients	Switzerland	20
2	Frontiers In Nutrition	Switzerland	6
3	Journal of Nutrition Health Aging	France	6
4	Current Nutrition Reports	USA	5
5	Alzheimers Dementia	USA	4
6	Circulation	USA	4
7	European Journal of Nutrition	Germany	4
8	JAPD-Journal of Prevention of Alzheimers Disease	France	4
9	Nutritional Neuroscience	England	4
10	American Journal of Clinical Nutrition	USA	3

### 3.3 Analysis of highly-cited publications

For the most high-cited publications, the first high-cited publication was conducted by Morris et al., published in *Alzheimers & Dementia* in 2015. In this study, they investigated the relationship between the risk of Alzheimer’s disease (AD) and the MIND diet, and it is the first study about the MIND diet. This study found that higher adherence to the MIND diet was associated with a lower risk of AD. They concluded that even modest dietary changes can have a substantial impact on reducing the risk of AD, emphasizing the role of dietary patterns in brain health and disease prevention. The second high-cited publication was conducted by Morris et al., published in Alzheimers & Dementia in 2015. It can be regarded as the sub-analysis of the above study. In this study, they found that the MIND diet could slow cognitive decline with age substantially. The third high-cited publication was published in Nutrients by Hellas Cena et al. in 2020. In this review, they discussed the benefits of some diets of MIND diet, DASH diet and Mediterranean diet on common non-communicable diseases (NCDs), such as cancer, CVDs, and diabetes. They concluded that MIND diet could reduce risks of NCDs of cancer and CVDs from clinical trials and epidemiological studies. The fourth high-cited publication was conducted by Brink et al., published in Advances in Nutrition in 2019. In this systematic review, they investigated the impacts of Mediterranean diet, MIND diet, and DASH diet on Alzheimer’s disease (AD), dementia, and cognitive decline. A total of 56 articles were included. They concluded that the strongest associations with a lower risk of AD and less cognitive decline were observed for the MIND diet. The fifth high-cited publication was published in Pharmacological Research by Pistollato et al. (10). In this review, they discussed the potential of dietary interventions such as MIND diet in managing and reducing the risk of AD and related non-psychiatric comorbidities. The attributes of the 20 most-cited documents are encapsulated in Table 3 (1, 2, 10–27).

**Table 3:** The top 20 most highly cited references on MIND diet.

Rank	Title	Journal	Citations	Year	First author
1	MIND diet associated with reduced incidence of Alzheimer’s disease	Alzheimers & Dementia	467	2015	Martha Clare Morris
2	MIND diet slows cognitive decline with aging	Alzheimers & Dementia	432	2015	Martha Clare Morris
3	Defining a Healthy Diet: Evidence for the Role of Contemporary Dietary Patterns in Health and Disease	Nutrients	301	2020	Hellas Cena
4	The Mediterranean, Dietary Approaches to Stop Hypertension (DASH), and Mediterranean-DASH Intervention for Neurodegenerative Delay (MIND) Diets Are Associated with Less Cognitive Decline and a Lower Risk of Alzheimer’s Disease-A Review	Advances In Nutrition	202	2019	Annelien C van den Brink
5	Nutritional patterns associated with the maintenance of neurocognitive functions and the risk of dementia and Alzheimer’s disease: A focus on human studies	Pharmacological Research	120	2018	Francesca Pistollato
6	MIND not Mediterranean diet related to 12-year incidence of cognitive impairment in an Australian longitudinal cohort study	Alzheimers & Dementia	107	2019	Diane E Hosking
7	Neuroprotective Diets Are Associated with Better Cognitive Function: The Health and Retirement Study	Journal of The American Geriatrics Society	85	2017	Claire T McEvoy
8	Association of long-term adherence to the mind diet with cognitive function and cognitive decline in American women	Journal of Nutrition Health & Aging	78	2018	A M Berendsen
9	The Nordic Prudent Diet Reduces Risk of Cognitive Decline in the Swedish Older Adults: A Population-Based Cohort Study	Nutrients	55	2018	Behnaz Shakersain
10	Nutrients in the Prevention of Alzheimer’s Disease	Oxidative Medicine And Cellular Longevity	52	2019	Anna Laura Cremonini
11	MIND Diet Associated with Reduced Incidence and Delayed Progression of Parkinsonism in Old Age	Journal of Nutrition Health & Aging	49	2018	P Agarwal
12	Does the MIND diet decrease depression risk? A comparison with Mediterranean diet in the SUN cohort	European Journal of Nutrition	42	2019	Ujué Fresán
13	Dietary pattern in relation to the risk of Alzheimer’s disease: a systematic review	Neurological Sciences	40	2019	Mehnoosh Samadi
14	Mediterranean-Dash Intervention for Neurodegenerative Delay (MIND) Diet Slows Cognitive Decline After Stroke	Journal of Prevention of Alzheimers Disease	39	2019	L Cherian
15	MIND and Mediterranean Diets Associated with Later Onset of Parkinson’s Disease	Movement Disorders	37	2021	Avril Metcalfe-Roach
16	The Role of Selected Bioactive Compounds in the Prevention of Alzheimer’s Disease	Antioxidants	36	2020	Wojciech Grodzicki
17	Mediterranean and MIND Diets Containing Olive Biophenols Reduces the Prevalence of Alzheimer’s Disease	International Journal of Molecular Sciences	36	2019	Syed Haris Omar
18	Mediterranean-DASH Intervention for Neurodegenerative Delay (MIND) study: Rationale, design and baseline characteristics of a randomized control trial of the MIND diet on cognitive decline	Contemporary Clinical Trials	35	2021	Xiaoran Liu
19	The role of diet in preventing and reducing cognitive decline	Current Opinion In Psychiatry	32	2020	Cristina Angeloni
20	Adherence to the MIND diet and prevalence of psychological disorders in adults	Journal of Affective Disorders	32	2019	Asma Salari-Moghaddam

### 3.4 Analysis of keywords

**Figure 5:**
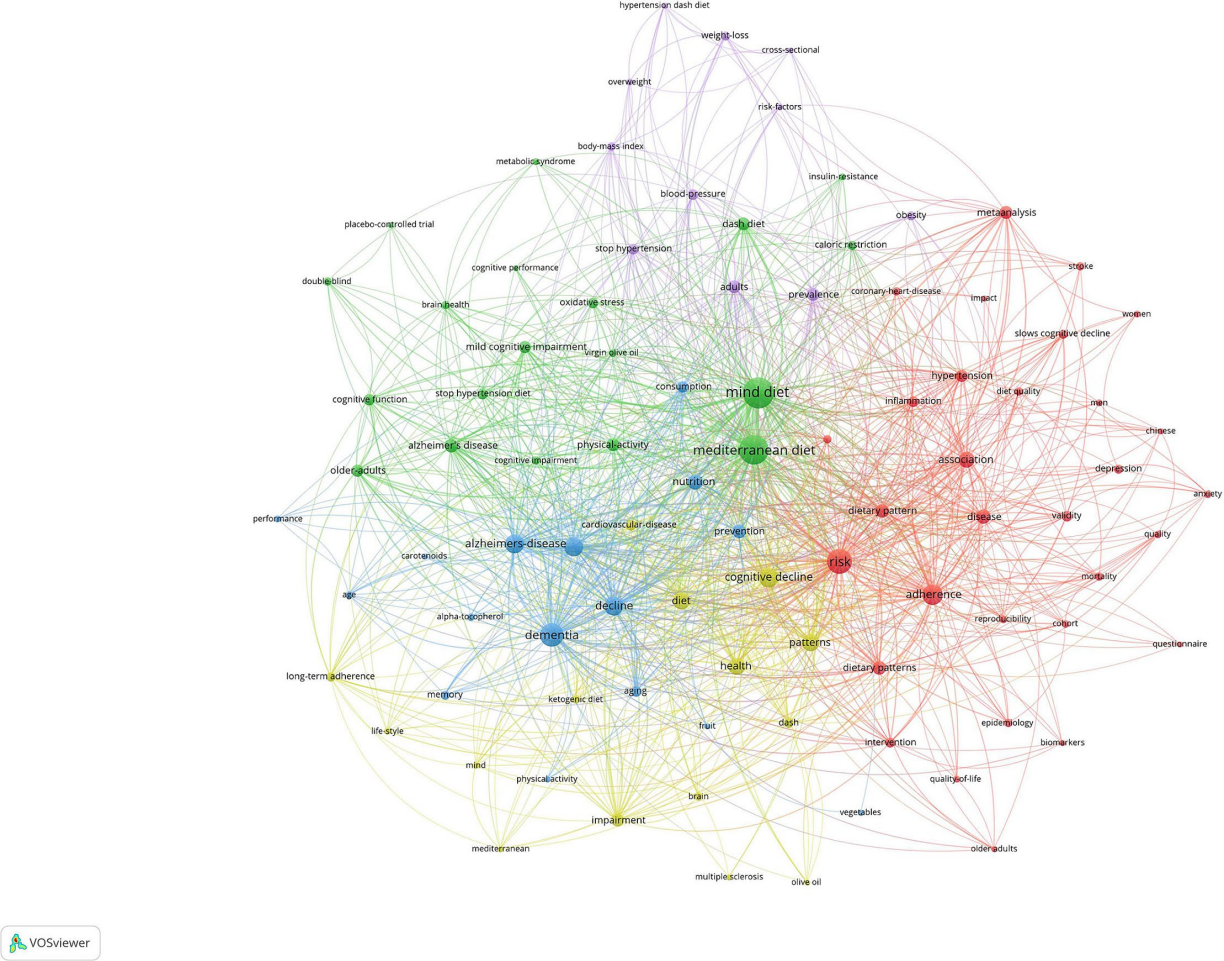
Visualization of keyword co-occurrence analysis on the MIND diet.

Figure 5 displayed the network visualization maps of co-occurrence of keywords, and the network visualization in the image reflects a bibliometric analysis of terms related to the MIND diet in scientific literature. Larger, centrally placed nodes such as “MIND diet,” “Mediterranean diet,” and “cognitive decline” indicate major areas of focus within the research field, with their size suggesting a higher prevalence of the term in the literature. The various colors of the nodes likely represent distinct thematic clusters, showing how topics are grouped within the research. The thickness of the lines connecting terms signifies the strength of the association between them, where thicker lines suggest a stronger relationship or more frequent co-occurrence in the literature. The network diagram illustrates the interconnectivity of dietary, cognitive, and health-related research topics, particularly highlighting the MIND diet’s role in studies pertaining to cognitive health and aging. The five clusters and key terms identified could be analyzed as follows: Dietary Interventions and Cognitive Health Cluster: A substantial cluster around the “MIND diet” is connected to “Alzheimer’s disease” and “cognitive decline,” indicating a primary research focus on dietary interventions for cognitive health and potentially slowing cognitive impairment. Nutritional Patterns and Health Effects Cluster: Proximal terms such as “diet,” “nutrition,” and “Mediterranean diet” suggest investigations into various dietary regimens and their broader implications for health, especially in the context of aging and cognitive function. Metabolic and Clinical Research Cluster: The presence of terms like “obesity,” “blood pressure,” and “body mass index” points to a concentration on the metabolic effects of diets and their clinical relevance, potentially in relation to cognitive outcomes. Health Outcomes and Lifestyle Factors: This part of the visualization includes terms like “physical activity,” “health,” and “lifestyle,” indicative of research on the outcomes of the MIND diet on overall health and factors related to lifestyle choices and behaviors. Emerging and Specialized Topics: Peripheral terms may represent niche areas or emerging topics within the field, such as “double-blind” and “placebo-controlled trial,” pointing to the methodologies used to assess the efficacy of dietary interventions on cognitive health. The network indicates that the MIND diet is extensively studied in relation to cognitive health and aging, with significant research interest in how dietary patterns affect various health outcomes and biological processes. The map reflects the multifaceted nature of research in this field, encompassing diet, disease, lifestyle factors, and their interrelationships, with a notable emphasis on the prevention and mitigation of cognitive decline through diet.

## 4 Discussion

To the best of our knowledge, this is the first comprehensive bibliometric analysis about MIND diet. The bibliometric analysis of the MIND diet reveals a growing body of research, with 171 documents identified in the study. This research is globally distributed, with contributions from 953 authors across 362 institutions in 37 countries/regions, and published in 94 journals. The United States leads with 72 publications, affirming its significant role in MIND diet research. Iran and the People’s Republic of China also show notable engagement with 28 and 19 publications, respectively, indicating a widespread international interest in the diet. Academically, Rush University stands out with 21 publications, followed by Harvard University and Tehran University of Medical Sciences, demonstrating their substantial contributions to this field. Martha Clare Morris is a key figure with 10 publications, alongside Klodian Dhana and Puja Agarwal, each contributing 9 publications, highlighting their influence in MIND diet research. The journal “Nutrients” is a major publication venue with 20 related articles, followed by “Frontiers in Nutrition” and “Journal of Nutrition Health Aging,” reflecting their crucial roles in advancing knowledge about the MIND diet. The first high-cited publication was conducted by Morris et al., published in *Alzheimers & Dementia* in 2015. In this study, they investigated the relationship between the risk of disease (AD) and the MIND diet, and it is the first study about the MIND diet. This study found that higher adherence to the MIND diet was associated with a lower risk of AD. In keyword analysis, terms like “MIND diet,” “Mediterranean diet,” and “cognitive decline” are prominent, indicating major research areas. The network visualization showcases the interconnectivity of dietary, cognitive, and health-related research, with a focus on dietary interventions for cognitive health and the role of dietary patterns in preventing neurodegenerative diseases. In summary, the MIND diet’s bibliometric analysis underscores its increasing scientific prominence, especially in the context of cognitive health and aging. The significant contributions from leading countries, institutions, and researchers highlight the MIND diet’s global relevance and the collaborative nature of the research, reflecting a comprehensive approach to understanding the diet’s impact on neurodegenerative disease prevention. The leadership of the USA, Rush University, and Martha Clare Morris in MIND diet research has catalyzed significant global shifts in both research priorities and health policies regarding dietary approaches to cognitive health. As the originator of the MIND diet, Martha Clare Morris, a nutritional epidemiologist at Rush University, leveraged a robust methodological approach to integrate elements of the Mediterranean and DASH diets, both already noted for their cardiovascular benefits, into a new regimen tailored specifically toward neuroprotection. The empirical evidence produced under her guidance, demonstrating the diet’s efficacy in reducing Alzheimer’s disease risk, not only validated the MIND diet but also set a precedent for dietary intervention in cognitive decline prevention. This pioneering work has prompted a broader adoption of preventative strategies in public health agendas worldwide, shifting focus from treatment to prevention. Research institutions and health policymakers globally are now increasingly prioritizing dietary research with similar preventive potential, recognizing the MIND diet’s implications for aging populations and the related economic and social benefits of enhancing cognitive health through accessible, non-pharmacological means. This shift underscores an evolving understanding that diet can significantly influence long-term health outcomes, encouraging further investigation and integration of dietary strategies into public health guidelines internationally.

### 4.1 The effects of the MIND diet for health outcomes

On the basis of publications, highly-cited publications, and important keywords with high frequency of MIND diet, the research hotspots were summarized as follows:

1. MIND diet for cognitive function (3, 12, 17, 18, 21, 28–35). MIND diet is proposed for dementia prevention and neuroprotection based on the dietary components and servings. A recent study (36) conducted by Yuan et al. investigated the impacts of MIND diet on dementia in three cohort populations with 18,136 participants, and then conducted a meta-analysis of 11 studies with 224,049 individuals (37–40), and they concluded that the MIND diet was related to lower risk of incident dementia in older and middle-aged participants. A study led by P. Agarwal and colleagues investigated the impact of the MIND diet on the incidence and progression of parkinsonism in older adults (21). They concluded that the MIND diet, specifically formulated to promote brain health, may be effective in decreasing the risk and slowing down the progression of parkinsonism in older adults. Although the results from these studies are optimistic, these studies are observational, and the conclusions are not confirmed by a recent study, which is the first interventional study to investigate the cognitive effects of the MIND diet in older adults (41). In this study, 604 older adults were enrolled. Compared with the control diet with mild caloric restriction, there were no statistically significant for changes in brain MRI and cognition outcomes in MIND diet group. The relatively short duration of the intervention and similar weight loss between the intervention and control groups might have contributed to the null findings. This highlights the urgency and necessity of further research to investigate the efficacy of MIND diet for cognitive function in interventional studies, especially in high-quality RCTs in particular on long-term outcomes.
2. MIND diet for other health outcomes (11, 15, 42–51), including CVDs, cancer, and diabetes. Akbar et al. (52) conducted a systematic review to investigate the relationship between the MIND diet and cardiometabolic diseases and risk factors, and they concluded that the MIND diet may be an effective dietary pattern to reduce cardiometabolic risk. Tison et al. (53) conducted a prospective study to explore the association between some diets of Mediterranean diet, MIND diet, and DASH diet and T2DM. 8,750 adults were included, and 1,026 cases of incident T2DM (11.7%) were occurred during follow-up. The results demonstrated that the MIND diet had strong association with lower T2DM incidence. Aghamohammadi et al. (42) performed a case–control study to examine the association between adherence to MIND diet and breast cancer, and the results showed that adherence to the MIND diet was associated with a lower risk of breast cancer.

The MIND diet has notably shifted global research priorities and health policy. Its empirical success in mitigating cognitive decline has prompted a broader reevaluation of the preventive potential of dietary strategies in public health. As such, research funding and initiatives increasingly prioritize nutritional studies that promise to delineate more effective preventive measures against cognitive diseases. This focus on non-pharmacological interventions, such as diet modification, not only caters to an aging population but also aligns with a global shift toward sustainable, cost-effective health solutions. Consequently, health policies around the world are beginning to incorporate guidelines that promote dietary patterns like those recommended by the MIND diet, recognizing their potential to significantly reduce healthcare costs and improve the quality of life for millions of elderly individuals. This integration into public health strategies underscores the pivotal role of diet in chronic disease prevention and fosters a more holistic approach to health and wellness globally. For further directions, despite substantial epidemiological data suggesting links between the MIND diet and neurodegenerative diseases, there is a significant lack of direct evidence demonstrating effects beyond those observed in confounded cohort studies. With only six randomized controlled trials (RCTs) among 171 studies, the evidence is insufficient to definitively recommend the MIND diet as a treatment option. New high-quality evidence should be provided in further studies.

There are some limitations in this study. Firstly, the overall number of documents related to the MIND diet remains relatively small. Furthermore, our analysis was confined to publications until October 15, 2023. They might introduce a degree of selection bias.

In conclusion, this is the first comprehensive bibliometric study that quantitatively and qualitatively analyzed the publications in the field of the MIND diet. The MIND diet may be a promising dietary pattern for dementia. However, the current evidence is restricted, and highlights the urgency and necessity of further research to investigate the efficacy of this diet for cognitive function. In addition, MIND diet may have some benefits for other health outcomes, including CVDs, cancer, and diabetes. The number of studies in the field of the MIND diet is limited. More studies are needed, and will give us more knowledge about the MIND diet to improve human health, especially for dementia.

## Data availability statement

The datasets presented in this study can be found in online repositories. The names of the repository/repositories and accession number(s) can be found in the article/supplementary material.

## Author contributions

LD: Writing – original draft, Writing – review & editing, Data curation, Methodology, Supervision, Conceptualization, Formal analysis, Project administration, Validation, Visualization, Software. XL: Conceptualization, Data curation, Investigation, Methodology, Software, Writing – original draft, Writing – review & editing. SW: Data curation, Formal analysis, Methodology, Project administration, Supervision, Writing – original draft, Writing – review & editing. YG: Writing – original draft, Writing – review & editing, Data curation, Supervision, Project administration, Resources, Visualization. FH: Writing – original draft, Writing – review & editing, Data curation, Supervision, Conceptualization, Formal analysis, Project administration, Validation, Investigation, Resources, Visualization.
